# Systematic review of Mendelian randomization studies on antihypertensive drugs

**DOI:** 10.1186/s12916-024-03760-x

**Published:** 2024-11-20

**Authors:** Bohan Fan, Junmeng Zhang, Jie V. Zhao

**Affiliations:** 1 School of Public Health, Li Ka Shing, Faculty of Medicine, The University of Hong Kong, Hong Kong, China; 2 State Key Laboratory of Pharmaceutical Biotechnology, The University of Hong Kong, Hong Kong SAR, China

**Keywords:** Antihypertensive drugs, ACE inhibitors, Calcium channel blockers, Beta-blockers, Systematic review, Mendelian randomization

## Abstract

**Background:**

We systematically reviewed Mendelian randomization (MR) studies and summarized evidence on the potential effects of different antihypertensive drugs on health.

**Methods:**

We searched PubMed and Embase for MR studies evaluating the effects of antihypertensive drug classes on health outcomes until 22 May 2024. We extracted data on study characteristics and findings, assessed study quality, and compared the evidence with that from randomized controlled trials (RCTs).

**Results:**

We identified 2643 studies in the search, of which 37 studies were included. These studies explored a wide range of health outcomes including cardiovascular diseases and their risk factors, psychiatric and neurodegenerative diseases, cancer, immune function and infection, and other outcomes. There is strong evidence supporting the protective effects of genetically proxied antihypertensive drugs on cardiovascular diseases. We found strong protective effects of angiotensin-converting enzyme (ACE) inhibitors on diabetes whereas beta-blockers showed adverse effects. ACE inhibitors might increase the risk of psoriasis, schizophrenia, and Alzheimer’s disease but did not affect COVID-19. There is strong evidence that ACE inhibitors and calcium channel blockers (CCBs) are beneficial for kidney and immune function, and CCBs showed a safe profile for disorders of pregnancy. Most studies have high quality. RCT evidence supports the beneficial effects of ACE inhibitors and CCBs on stroke, diabetes, and kidney function. However, there is a lack of reliable RCTs to confirm the associations with other diseases.

**Conclusions:**

Evidence of the benefits and off-target effects of antihypertensive drugs contribute to clinical decision-making, pharmacovigilance, and the identification of drug repurposing opportunities.

**Supplementary Information:**

The online version contains supplementary material available at 10.1186/s12916-024-03760-x.

## Background

Hypertension, which affects more than one billion adults globally, is a major risk factor of heart, brain, kidney, and other chronic diseases [1]. In addition to the cardiovascular benefits, antihypertensive drugs may also affect cancer [2], inflammation [3], kidney disease [4], and mental disorders [5]. Different classes of antihypertensive drugs have distinct mechanisms of action and may exhibit different off-target effects. For example, angiotensin-converting enzyme (ACE) inhibitors and calcium channel blockers (CCBs) have shown benefits for kidney function, whereas beta-blockers (BBs) have not demonstrated such benefits [6]. Given hypertension requires lifelong medication use, understanding the potential effects associated with different classes of antihypertensive drugs is crucial for patients with hypertension and other comorbidities. Previous observational studies have examined the effects of antihypertensives on various health outcomes [7, 8], for example, one recent study explored the effects on 262 outcomes using a target trial emulation approach [7]. However, observational studies are open to residual confounding by socioeconomic position and environmental factors, which cannot establish causality.

Randomized controlled trials (RCTs) are regarded as the “gold standard” to evaluate drug safety and effectiveness but not for assessing off-target effects. By assigning participants randomly to a treatment or control group, RCT allows the estimation of drug effectiveness without confounding and selection bias that are often presented in real-world studies [9]. However, RCTs are expensive, time-consuming, and sometimes not feasible or ethical to conduct. Mendelian randomization (MR) study, also known as “nature’s randomized trials,” is an instrumental variable analysis for causal inference using observational data [9]. It utilizes genetic variants randomly assigned at birth as instruments, which means that it is not affected by socioeconomic position and therefore minimizes confounding [10]. Drug-target MR has been recommended to be used to examine the efficacy and off-target effects of drugs [11], and has been widely used in different drugs [12–14], including antihypertensive drugs [6, 13, 15–17]. By choosing genetic instruments strongly associated with the exposure from a drug target gene region, drug-target MR provides an alternative source of evidence on the unexpected adverse events [18]. For example, MR on statins results showed statins have off-target effects of increasing the risk of diabetes [19], similar to RCTs [20]. Here, we carried out a systematic review to comprehensively summarize and appraise the evidence from MR studies on the causal relationships of antihypertensive drug classes with different disease outcomes.

## Methods

This systematic review was conducted following the Preferred Reporting Items for Systematic Reviews and Meta-Analysis (PRISMA) guideline [21]. The study protocol was registered at PROSPERO, with registration number CRD42022360602.

### Search strategy

This systematic review included MR studies that assessed the effects of different classes of antihypertensive drugs, including ACE inhibitors, angiotensin receptor blockers (ARBs), CCBs, alpha-adrenoceptor blockers, adrenergic neuron blocking drugs, BBs, centrally acting antihypertensive drugs, loop diuretics, potassium-sparing diuretics and aldosterone antagonists, renin inhibitors, thiazides and related diuretics, and vasodilator antihypertensives on all health outcomes. Two independent reviewers (B.H.F and J.M.Z) searched PubMed and Embase databases for published MR studies in English until 22 May 2024 using a combination of key terms such as “Mendelian randomization,” “antihypertensive drugs,” and their synonyms. The detailed search strategy is shown in Additional file 1: Methods. After removing duplicates, we screened the titles and abstracts of studies to determine their eligibility based on inclusion and exclusion criteria and then reviewed full texts. The search was complemented by checking the reference lists of the included studies. Disagreements between reviewers were resolved by discussion with a third reviewer (J.V.Z).

### Inclusion–exclusion criteria

We included original MR studies that evaluated the association of antihypertensive drugs with health outcomes. We excluded studies that (i) employed a systematic screening method to identify risk factors associated with outcomes or included antihypertensive drugs as one of multiple exposures; (ii) used antihypertensive drugs intake, rather than genetic proxies for antihypertensive drugs, as exposure; (iii) only considered the combined effect of antihypertensive drugs; (iv) only considered gene-specific effects for drugs rather than the effects of antihypertensive drug classes; (v) were not MR studies; or (vi) were not published original studies such as conference abstracts and preprints, reviews, letters, short communications, commentaries, editorials, study proposals, or methodological papers.

### Data extraction

B.H.F extracted information on the publication details (title, first author’s name, publication year, PMID identifier), exposure (antihypertensive drugs class, unit, exposure data source, exposure study population), outcome (diseases or biomarkers, sample size, outcome data source, outcome study population), study design (one/two-sample), main analysis method, effect sizes with 95% confidence interval (CI) and *p*-values reported in abstracts, and sensitivity analyses conducted, such as MR-Egger regression, weighted median, weighted mode, Mendelian Randomization Pleiotropy RESidual Sum and Outlier (MRPRESSO), and multivariable MR. J.M.Z verified the accuracy and completeness of the extracted data.

### Strength of evidence

We categorized the strength of evidence into three levels: strong, suggestive, and unclear evidence (Additional file 2: Table S1), based on the results of the main analysis, their consistency with the results of the sensitivity analysis, as well as study quality. If an association is found to be significant after multiple testing and remains consistent with the directions of associations in the sensitivity analysis, we consider it as “strong evidence.” If an association is significant after multiple testing correction but does not align with the direction of associations in the sensitivity analysis, or if an association is insignificant after multiple testing but still has a *p*-value < 0.05 (known as nominal association) and is directionally consistent with the sensitivity analysis results, we consider it as “suggestive evidence.” In cases where there is only a nominal association and it is inconsistent with the sensitivity analysis or the study power < 80%, we consider it as “unclear evidence.” To take into account of study quality, we also incorporated the study quality assessment, for example, the strength of evidence of a study is downgraded when the quality is low-to-moderate.

### Risk-of-bias assessment

We designed risk assessment criteria for MR studies based on the guideline for strengthening the reporting of observational studies in epidemiology using Mendelian randomization (STROBE-MR) and three key MR assumptions [10, 22]. We assigned a score of "1" if the requirement was adequately met, and a score of “0” if it was not. A detailed grading scheme is provided in Additional file 2: Table S2.

The “Relevance” assumption fulfillment was evaluated based on the following criteria: (1) whether the genetic association of the instruments was strongly associated with exposure (i.e., *p* < 5 × 10^−8^); and (2) whether the *F*-statistic of the genetic instruments was above 10. For the “Independence” assumption, we assessed (3) whether the study examined the genetic association with potential confounders using PhenoScanner or used multivariable MR analysis to address potential pleiotropy; and (4) whether the study controlled for population stratification or used ethnically homogenous populations in the analysis. We assessed whether the study achieved the “Exclusion-restriction” assumption by checking (5) whether the study conducted sensitivity analyses using various MR methods, such as weighted median, weighted mode, MR-Egger regression with pleiotropy test, MRPRESSO, or multivariable MR; and (6) whether the study assessed possibility of pleiotropy using Bayesian colocalization analysis, control outcomes, or conducting replication using another set of instruments or validation cohorts. Additionally, we commented on the statistical power of each study to ensure that adequate sample sizes were utilized for causal inference. We also compared the results from MR with those of RCTs to strengthen the conclusions.

## Results

**Fig. 1:**
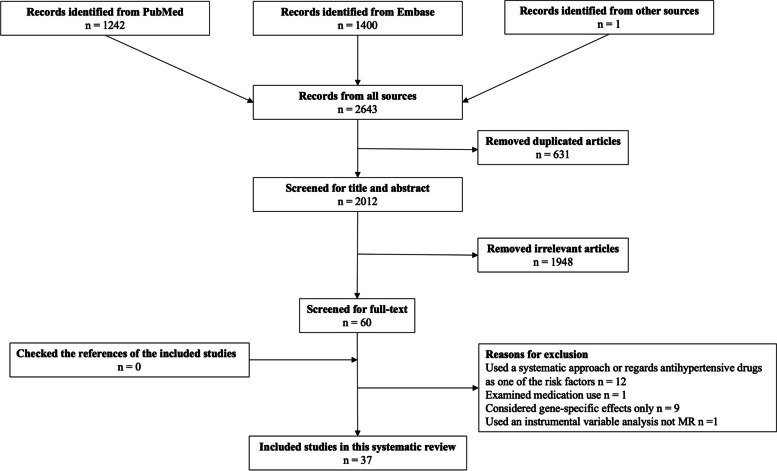
Flow chart of study selection process

A total of 2643 potentially eligible studies were identified, 631 were excluded due to duplicate, and 1948 irrelevant articles were excluded after screening on title and/or abstract. After full-text review, 23 were removed and 37 studies remained (Fig. 1).

### Study characteristics

The included studies assessed different outcomes, including circulatory diseases (*n* = 10), type 2 diabetes, lipid and glycaemic traits (*n* = 2), renal function and kidney stones (*n* = 2), psychiatric disorders (*n* = 1), neurological diseases (*n* = 6), cancer (*n* = 4), infectious disease (*n* = 2), immune function (*n* = 1), glaucoma (*n* = 1), psoriasis (*n* = 2), musculoskeletal health and geriatric conditions (*n* = 3), longevity (*n* = 1), erectile dysfunction (*n* = 1) as well as disorders during pregnancy (*n* = 1). Study characteristics and all extracted MR results are summarized in Additional file 2: Table S3.

### Main findings

**Fig. 2:**
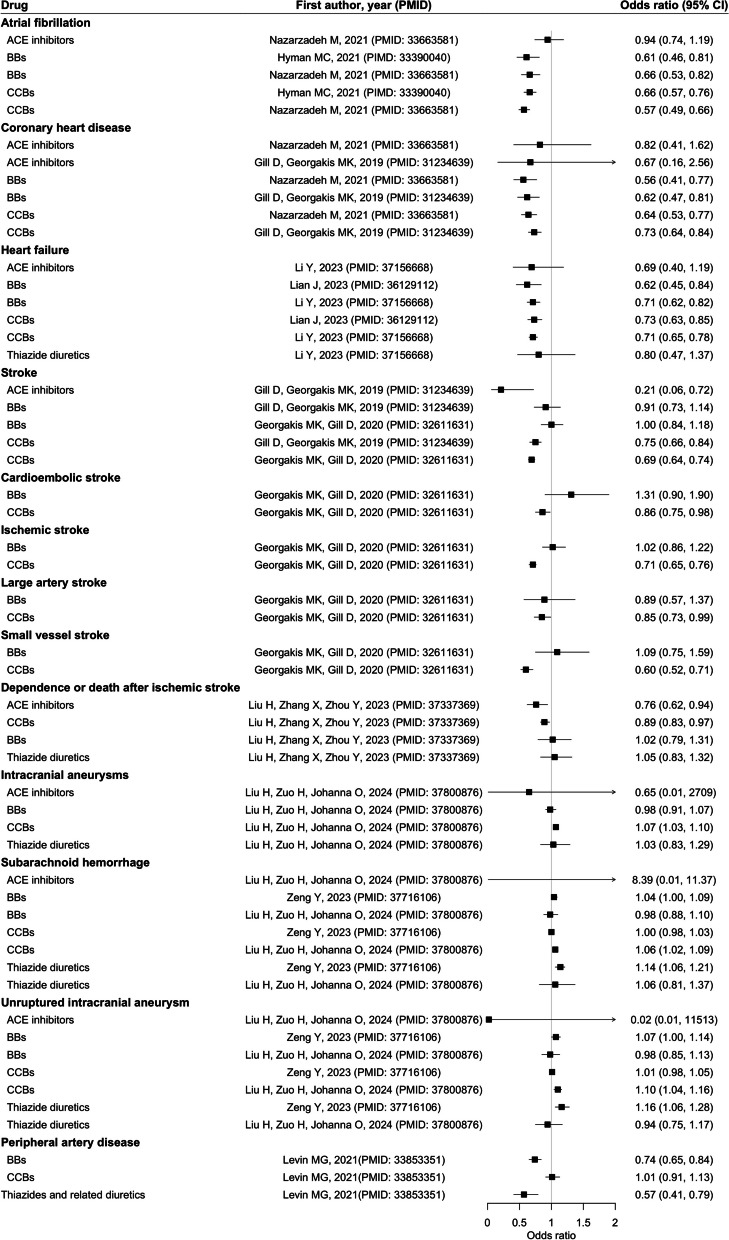
Results of major antihypertensive drugs and circulatory diseases. Units of exposure are obtained from the original studies, as shown in Table S3. Abbreviations: angiotensin-converting enzyme (ACE); beta-adrenoceptor blockers (BBs); calcium channel blockers (CCBs)

**Fig. 3:**
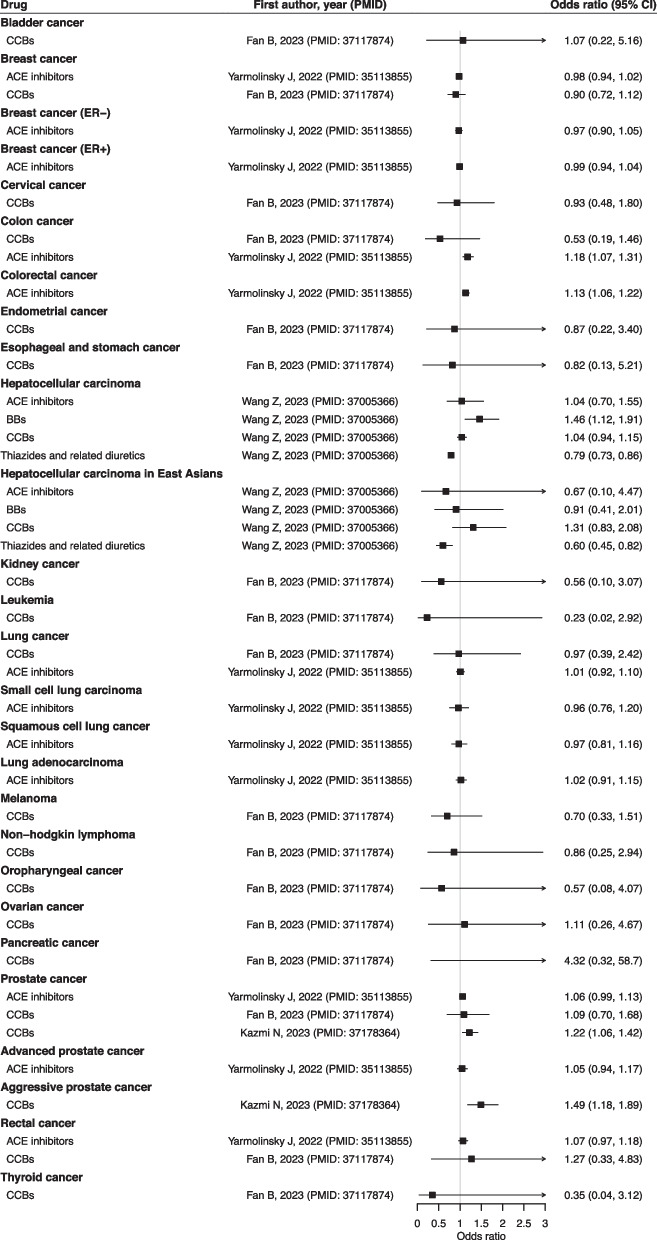
Results of major antihypertensive drugs and cancer. Units of exposure are obtained from the original studies, as shown in Table S3. Abbreviations: angiotensin-converting enzyme (ACE); beta-adrenoceptor blockers (BBs); calcium channel blockers (CCBs)

**Fig. 4:**
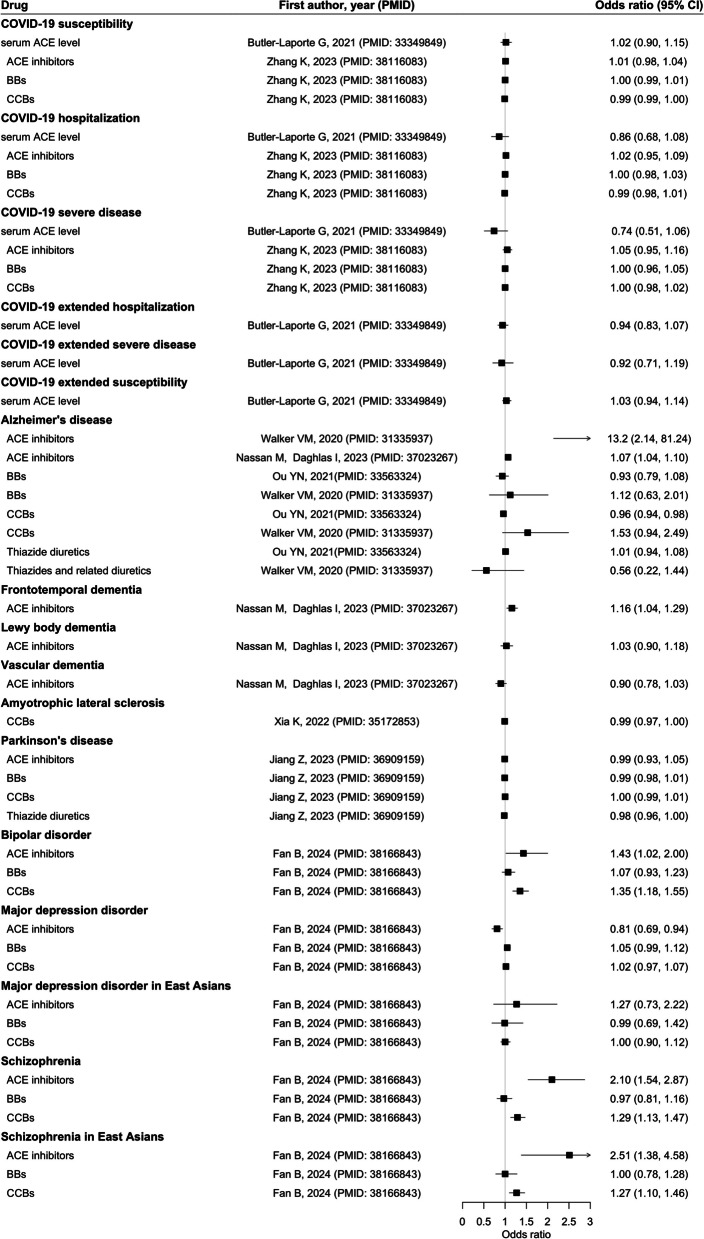
Results of major antihypertensive drugs and infectious diseases, mental and neurological disorders. Units of exposure are obtained from the original studies, as shown in Table S3. Angiotensin-converting enzyme (ACE); beta-adrenoceptor blockers (BBs); calcium channel blockers (CCBs)

**Fig. 5:**
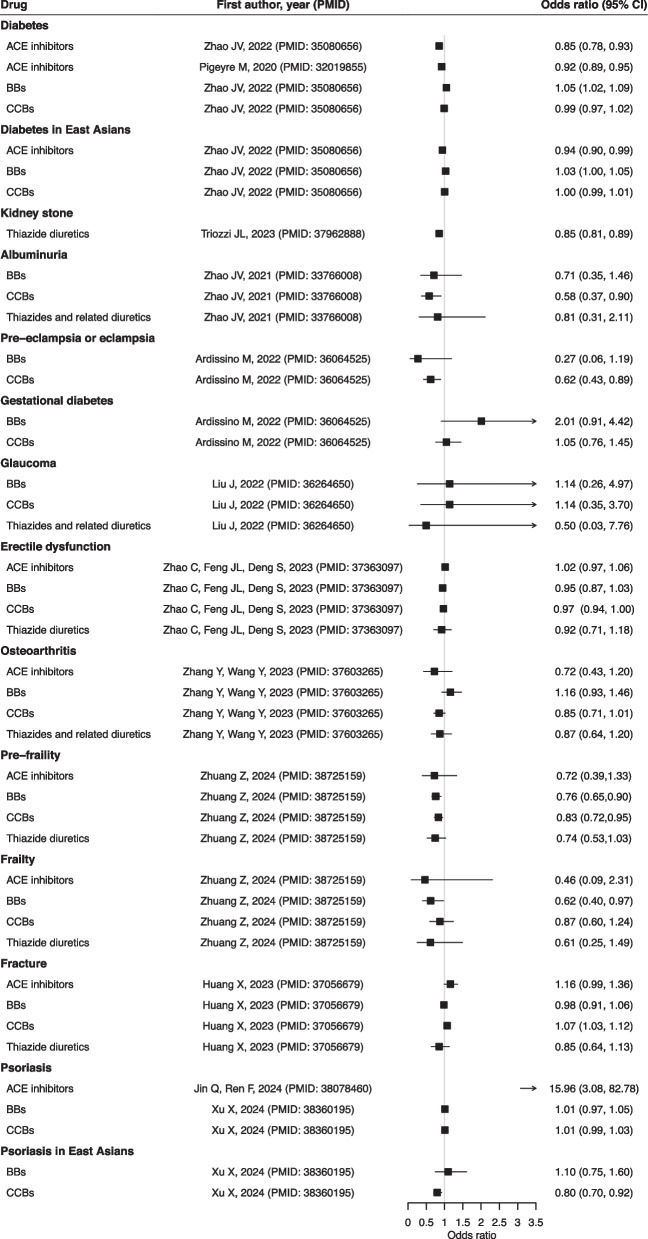
Results of major antihypertensive drugs and other diseases. Units of exposure are obtained from the original studies, as shown in Table S3. Angiotensin-converting enzyme (ACE); beta-adrenoceptor blockers (BBs); calcium channel blockers (CCBs)

The forest plots showed the associations between frequently investigated antihypertensive drugs, ACE inhibitors, BBs, CCBs, and thiazide diuretics, with diseases (Figs. 2, 3, 4, and 5) and biomarkers (Additional file 3: Fig. S1), as well as the findings for other antihypertensives (Additional file 3: Fig. S2). The strength of evidence is summarized in Additional file 2: Table S4. For circulatory diseases, ACE inhibitors and CCBs were associated with a lower risk of stroke [13, 23], and a better functional outcome after ischemic stroke [24]. Genetically proxied BBs and CCBs were consistently associated with lower risk of coronary heart disease (CHD) [13, 16], atrial fibrillation [15, 16], and heart failure [25, 26]. Genetically proxied CCBs might increase the risk of intracranial aneurysms and subarachnoid hemorrhage [27] while thiazide diuretics could lower their risks [28]. Genetically proxied BBs, loop diuretics, and thiazide diuretics could possibly reduce the risk of peripheral artery disease [17].

For kidney health, one study found that genetically proxied ACE inhibitors and CCBs showed possible protective effects as ACE inhibitors were linked to increased estimated glomerular filtration rate and CCBs were associated with lower urine albumin-to-creatinine ratio and a lower risk of albuminuria, whereas genetic proxied BBs were associated with lower estimated glomerular filtration rate [6]. Another study suggested that genetic proxies for thiazide diuretics were associated with a lower risk of kidney stones [29]. Regarding diabetes and metabolic disorders, two studies indicated that genetically proxied ACE inhibitors may lower the risk of type 2 diabetes [30, 31], while BBs showed an inverse association in both European and East Asian populations [31]. In addition, genetically proxied BBs were associated with lower levels of high-density lipoprotein cholesterol and higher levels of triacylglycerols while genetically proxied CCBs were associated with higher levels of low-density lipoprotein cholesterol [31].

Regarding mental and neurological diseases, ACE inhibitors were possibly related to a higher risk of schizophrenia in European and East Asian populations [32]. ACE inhibitors may be linked to increased risk of Alzheimer’s disease (AD) and frontotemporal dementia [33] while CCBs may be associated with a lower risk of AD [34]. However, one study reported limited evidence that antihypertensive drug classes would affect the risk of AD [35]. Genetically proxied CCBs were related to a lower risk of amyotrophic lateral sclerosis [36] and an earlier onset age of Huntington disease [37]. However, antihypertensive drugs were unlikely to have an impact on the risk of Parkinson’s disease or its age at onset [38].

In terms of cancer, genetically proxied ACE inhibitors were found to increase the risk of colorectal cancer, but they did not appear to affect the risk of breast cancer, lung cancer, or prostate cancer [39]. Null associations were observed between genetic proxies for CCBs with different cancers including non-Hodgkin lymphoma, melanoma, leukemia, thyroid, rectal, pancreatic, oral cavity/pharyngeal, kidney, stomach, colon, bladder, endometrial, cervical and breast, lung and ovarian cancer [40]. However, it is possible that CCBs may increase the risk of prostate cancer [41]. Genetically proxied thiazides and related diuretics were protective against hepatocellular carcinoma while genetically proxied BBs may increase its risk [42].

ACE inhibitors showed no association with COVID-19 susceptibility, hospitalization, or severity [43, 44]; however, a nominal negative association between genetically proxied CCBs and COVID-19 susceptibility was reported [44]. Regarding immune biomarkers, genetically proxied ACE inhibitors and CCBs were associated with increased lymphocyte and lower neutrophil percentage [45]. Genetically proxied ACE inhibitors may reduce TNF-alpha, whereas other drug classes had no effect on immune function or TNF-alpha [45].

MR studies also investigated the effects on other diseases. Antihypertensive drugs were not associated with glaucoma [46] or erectile dysfunction [47]. ACE inhibitors and loop diuretics could increase the risk of psoriasis [48, 49]. ARBs and thiazide diuretics were protective on bone mineral density while CCBs and potassium-sparing diuretics may have a negative effect [50]. CCBs and BBs were associated with reduced pre-frailty risk [51], and potassium-sparing diuretics and aldosterone antagonists showed a reduced risk of osteoarthritis [52]. Genetically proxied BBs, CCBs, and vasodilators may increase lifespan [53]. Genetically proxied CCBs were associated with a lower risk of pre-eclampsia but the effect was not shown in BBs [54]. Genetically proxied BBs were found to potentially increase the risk of having low-birthweight child [54].

### Risk-of-bias assessment

The risk-of-bias assessment of this review rated 33 studies as “high quality” (with a score no less than 4) and 4 studies as low-to-moderate quality (Additional file 2: Table S5). In the assessment of assumption 1 (“relevance”), 29 studies (78%) used genetic instruments strongly associated with exposure and with *F*-statistics > 10. In the assessment of assumption 2 (“independence”), 24 studies (65%) both controlled for population stratification and checked the genetic associations with potential confounders of exposure-outcome relationship or applied multivariable MR analysis. In the assessment of assumption 3 (“exclusion-restriction”), 26 studies (70%) used different MR analytic methods, such as weighted median, weighted mode, or multivariable MR analysis, and used other approaches to control for pleiotropy, such as control outcomes or conducting replication with another set of instruments. For power calculation, 7 (19%) performed power calculation with detailed interpretation, 19 (51%) considered the statistical power issue but did not perform a power calculation, and 11 (30%) did not perform or mention study power.

### Comparison with RCTs

Here, we compared RCTs and MR evidence in Table 1. Consistent RCTs and MR evidence showed protective roles of ACE inhibitors and CCBs in stroke [13, 23, 55–59], kidney function [6, 60, 61], and diabetes [30, 31, 62], and showed potential harmful effects of BBs on diabetes [30, 31, 60–62]. MR showed CCBs might increase the risk of intracranial aneurysms and subarachnoid hemorrhage, in contrast, RCTs showed that CCBs were beneficial for patients with subarachnoid hemorrhage by reducing the risk of poor outcome and secondary ischemia after aneurysmal subarachnoid hemorrhage [63]. MR studies have reported that ACE inhibitors may increase the risk of colorectal cancer [39], BBs may increase the risk of hepatocellular carcinoma [42], and CCBs may increase the risk of prostate cancer [41], but meta-analysis of RCTs generally reported null association of different classes of antihypertensive drugs with different types of cancer [55, 64]. Both MR and RCTs showed ACE inhibitors, BBs and CCBs were unlikely to exert effects on COVID-19 outcomes [65, 66] or erectile dysfunction [67]. MR and RCTs also consistently showed a protective effect of thiazide diuretics on bone mineral density [68]. We did not find high-quality RCTs on heart failure, psychiatric disorders, neurodegenerative disorders, glaucoma, psoriasis, immune function, frailty risk, and lifespan, so we cannot compare the evidence from MR and RCTs on these outcomes.

**Table 1:** Comparison between Mendelian randomization findings in this systematic review and findings from randomized controlled trials and their meta-analyses

Study	Exposure(s)	Outcome(s)	Mendelian randomization findings	Findings from randomized controlled trials and their meta-analyses
Circulatory diseases				
Gill D, Georgakis MK, 2019 (PMID: 31234639)	ACE inhibitors; BBs; CCBs	Stroke; coronary heart disease (CHD)	ACE inhibitors showed a protective effect against stroke but not CHD risk. BBs showed a protective effect against CHD but not stroke risk. CCBs showed a protective effect against both CHD and stroke risk	A systematic review and meta-analysis of randomized controlled trials (RCTs) reported that ACE inhibitors reduce the risk of stroke and CHD [55] A systematic review and meta-analysis of RCTs reported that CCBs are more effective than BBs in stroke prevention [56–58] Another systematic review and meta-analysis of RCTs found that antihypertensive drugs, in general, are protective against the progression of cerebral small vessel disease and white matter hyperintensities [59]
Georgakis MK, Gill D, 2020 (PMID: 32,611,631)	BBs; CCBs	Any stroke; ischemic stroke; large artery stroke; cardioembolic stroke; small vessel stroke; white matter hyperintensities	CCBs showed a protective effect against any stroke, ischemic stroke, and its subtypes (large artery, cardioembolic, small vessel stroke), as well as a reduction in white matter hyperintensities volume. However, BBs did not show any protective effects	
Liu H, Zhang X, Zhou Y, 2023 (PMID: 37337369)	ACE inhibitors; ARBs; BBs; CCBs; thiazide diuretics	Risk of dependence or death after ischemic stroke in 3 months	ACE inhibitors and CCBs showed a protective effect on functional outcomes after ischemic stroke, but BBs, ARBs, and thiazides did not show similar benefits	
Liu H, Zuo H, Johanna O, 2024 (PMID: 37800876)	CCBs	Intracranial aneurysms (IA); subarachnoid hemorrhage (SAH)	CCBs might increase the risk of IA and SAH	A systematic review and meta-analysis of RCTs demonstrated that CCBs are beneficial for patients with SAH, reducing the risk of adverse outcomes and secondary ischemia following aneurysmal SAH [63]
Zeng Y, 2023 (PMID: 37716106)	BBs; CCBs; thiazide diuretics	IA (non-ruptured); SAH	Thiazide diuretics showed a protective effect against the risk of IA (non-ruptured) and SAH, while BBs and CCBs showed no association with IA	RCTs are not available
Nazarzadeh M, 2021 (PMID: 33663581)	ACE inhibitors; BBs; CCBs	Atrial fibrillation; CHD	BBs and CCBs showed a protective effect against atrial fibrillation and CHD risk, but ACE inhibitors did not show any benefits	A systematic review and meta-analysis of RCTs found that antihypertensive drugs can reduce the risk of atrial fibrillation, with greater benefits in patients with heart failure. However, there is insufficient evidence to support similar benefits in patients without heart failure [69]
Hyman MC, 2021 (PMID: 33390040)	BBs; CCBs	Atrial fibrillation	BBs and CCBs showed a protective effect against the risk of atrial fibrillation	
Levin MG, 2021 (PMID: 33853351)	Alpha-adrenoceptor blockers; BBs; CCBs; loop diuretics; renin inhibitors; thiazide diuretics; vasodilators	Peripheral artery disease	BBs, loop diuretics, and thiazide diuretics showed protective effects on peripheral artery disease	A review of RCTs concluded insufficient evidence regarding the use of different antihypertensive drugs in individuals with peripheral artery disease [70]
Lian J, 2023 (PMID: 36129112)	ACE inhibitors; ARBs; BBs; CCBs; thiazide diuretics	Heart failure	BBs and CCBs showed a protective effect against heart failure risk, while no association was observed for ARBs	Some previous RCTs have investigated the effects of antihypertensive drug classes on preventing heart failure, but the results have been inconsistent and generally of low quality [57, 71–73]
Li Y, 2023 (PMID: 37156668)	ACE inhibitors; ARBs; BBs; CCBs; thiazide diuretics	Heart failure	BBs and CCBs showed a protective effect against the risk of heart failure	
Renal and kidney health				
Zhao JV, 2021 (PMID: 33,766,008)	ACE inhibitors; adrenergic neuron blocking drugs; alpha-adrenoceptor blockers; ARBs; BBs; CCBs; centrally acting antihypertensives; loop diuretics; potassium-sparing diuretics (PSDs); renin inhibitors; thiazides diuretics; vasodilators	Estimated glomerular filtration rate (eGFR); albuminuria; urine albumin-to-creatinine ratio	ACE inhibitors were linked to higher eGFR, while BBs were associated with lower eGFR. CCBs showed a protective effect on the risk of albuminuria and lower urine albumin-to-creatinine ratio	Systematic reviews and meta-analyses of RCTs found a consistent protective effect of ACE inhibitors and CCBs on kidney health [60, 61]
Triozzi JL, 2023 (PMID: 37962888)	Thiazide diuretics	Kidney stone	Thiazide diuretics showed a protective effect against the risk of kidney stones	An RCT reported a null association of thiazide diuretic (hydrochlorothiazide) with the recurrence of kidney stones [74]
Diabetes and metabolic disorders				
Zhao JV, 2022 (PMID: 35080656)	ACE inhibitors; BBs; CCBs	Diabetes; glucose; HbA1c; low-density lipoprotein (LDL)-cholesterol; High-density lipoprotein (HDL)-cholesterol; triacylglycerols	ACE inhibitors showed a protective effect against the risk of diabetes and HbA1c levels. In contrast, BBs were associated with a higher risk of diabetes, lower HDL-cholesterol and higher triacylglycerols. CCBs were associated with higher LDL-cholesterol	A systematic review and meta-analyses of RCTs showed that ACE inhibitors reduce the risk of new-onset type 2 diabetes; the use of BBs may increase this risk, while no significant effect was observed for CCBs [62]
Pigeyre M, 2020 (PMID: 32019855)	ACE inhibitors	Diabetes	ACE inhibitors showed a protective effect against diabetes risk	
Mental and neurological disorders				
Fan B, 2024 (PMID: 38166843)	ACE inhibitors; BBs; CCBs	Schizophrenia; bipolar disorder; major depressive disorder	ACE inhibitors were associated with an increased risk of schizophrenia in Europeans and East Asians. BBs were not associated with any mental disorders in Europeans and East Asians. CCBs showed no benefits on mental disorders	RCTs are not available
Walker VM, 2020 (PMID: 31335937)	ACE inhibitors; Adrenergic neuron blockers; Alpha-adrenoceptor blockers; ARBs; BBs; CCBs; centrally acting antihypertensives; loop diuretics; PSDs; renin inhibitors; thiazide diuretics; vasodilators	Alzheimer’s disease (AD)	The examined antihypertensive drugs did not affect AD risk via lowering systolic blood pressure	Systematic reviews and meta-analyses of RCTs indicated a lack of high-quality RCTs on AD, with limited evidence that antihypertensive treatment may reduce cognitive decline [75–78]
Ou YN, 2021 (PMID: 33,563,324)	ARBs; BBs; CCBs; thiazide diuretics	AD	CCBs showed a protective effect against AD risk	
Nassan M, Daghlas I, 2023 (PMID: 37023267)	ACE inhibitors	AD; frontotemporal dementia; lewy body dementia; vascular dementia	ACE inhibitors were associated with increased risks of AD and frontotemporal dementia	
Xia K, 2022 (PMID: 35172853)	CCBs	Amyotrophic lateral sclerosis	CCBs may lower the risk of amyotrophic lateral sclerosis	RCTs are not available
Zhu Y, 2023 (PMID: 37226269)	ACE inhibitors; BBs; CCBs	Age at onset of Huntington’s disease	CCBs were associated with an earlier age at onset of Huntington’s disease, while BBs and ACE inhibitors were not associated with these outcomes	RCTs are not available
Jiang Z, 2023 (PMID: 36909159)	ACE inhibitors; ARBs; BBs; CCBs; thiazide diuretics	Parkinson’s disease risk and its age at onset	ACE inhibitors, ARBs, BBs, CCBs, and thiazide diuretics were not associated with the risk of Parkinson’s disease	RCTs are not available
Cancer				
Yarmolinsky J, 2022 (PMID: 35113855)	ACE inhibitors	Overall and subtype-specific cancers: breast cancer; colorectal cancer; lung cancer; prostate cancer	ACE inhibition was associated with increased risk of colorectal cancer, but not breast cancer, lung cancer, or prostate cancer	Two systematic reviews and meta-analyses of RCTs reported null associations between antihypertensive drugs and cancer risk [55, 64]
Fan B, 2023 (PMID: 37117874)	CCBs	17 cancers	Null associations were observed for CCBs with non-Hodgkin lymphoma, melanoma, leukemia, thyroid, rectal, pancreatic, oral cavity/pharyngeal, kidney, esophagus/stomach, colon, bladder, endometrial, cervical, breast, prostate, lung, and ovarian cancer	
Wang Z, 2023 (PMID: 37005366)	ACE inhibitors; ARBs; BBs; CCBs; centrally acting antihypertensives; loop diuretics; PSDs; renin inhibitors; thiazide diuretics; vasodilators	Hepatocellular carcinoma	Thiazides and related diuretics were associated with decreased risk of hepatocellular carcinoma in both Europeans and East Asians while BBs were strongly associated with increased risk of hepatocellular carcinoma in Europeans	
Kazmi N, 2023 (PMID: 37178364)	CCBs	Prostate cancer	CCBs were associated with increased risk of overall prostate cancer and aggressive prostate cancer	
Infectious diseases				
Butler-Laporte G, 2021 (PMID: 33349849)	ACE inhibitors	COVID-19 susceptibility, extended susceptibility, hospitalization, extended hospitalized, severity, extended severe disease	Serum ACE levels were not associated with COVID-19 susceptibility, hospitalization, or severity	Systematic reviews and meta-analyses of RCTs showed null associations of ACE inhibitors and CCBs with the risk, severity, and negative outcomes of COVID-19 [65, 66]
Zhang K, 2023 (PMID: 38116083)	ACE inhibitors; BBs; CCBs	COVID-19 susceptibility, hospitalization, severity	ACE inhibitors and BBs were not associated with COVID-19 risks but CCBs were nominally associated with a reduced susceptibility to COVID-19	
Immune biomarkers				
Zhao JV, 2021 (PMID: 33025652)	ACE inhibitors; Adrenergic neuron blocking drugs; alpha-adrenoceptor blockers; ARBs; BBs; CCBs; centrally acting antihypertensives; loop diuretics; PSDs; renin inhibitors; thiazide diuretics; vasodilators	Lymphocyte; neutrophil; tumor necrosis factor-alpha (TNF-alpha)	ACE inhibitors were associated with increased lymphocyte percentage, decreased neutrophil percentage, and possibly lowered TNF-alpha. CCBs, PSDs, aldosterone antagonists, and vasodilator antihypertensives showed a similar effect on immune markers, lymphocyte and neutrophil percentages, but were not related to TNF-alpha. Other classes of hypertensives, including ARBs, had no effect on immune markers or TNF-alpha	RCTs are not available
Ophthalmic diseases				
Liu J, 2022 (PMID: 36264650)	ACE inhibitors; ARBs; BBs; CCBs; centrally acting antihypertensives; loop diuretics; PSDs; renin inhibitors; thiazide diuretics; vasodilators	Glaucoma	The examined antihypertensive drugs were not associated with glaucoma	RCTs are not available
Dermatological diseases				
Jin Q, Ren F, 2024 (PMID: 38078460)	ACE inhibitors	Psoriasis	ACE inhibitors were probably associated with an increased the risk of psoriasis	RCTs are not available
Xu X, 2024 (PMID: 38360195)	BBs; CCBs; loop diuretics; vasodilators	Psoriasis	Loop diuretics may be associated with an increased risk of psoriasis in Europeans, but a decreased risk in East Asians. CCBs showed a protective effect against the risk of psoriasis in East Asians	
Musculoskeletal health and geriatric conditions				
Huang X, 2023 (PMID: 37056679)	ACE inhibitors; alpha-blockers; ARBs; BBs; CCBs; loop diuretics; PSDs; thiazide diuretics	Fracture; total body bone mineral density (TB-BMD); estimated heel bone mineral density (eBMD)	ARBs and thiazide diuretics may have a protective effect on bone health, as ARBs were associated with a reduced risk of fracture, higher TB-BMD, and higher eBMD, and thiazide diuretics showed positive associations with eBMD. In contrast, CCBs and PSDs may have a negative effect; CCBs were linked to an increased risk of fracture, while PSDs showed negative associations with TB-BMD	Two RCTs showed that the thiazide diuretic hydrochlorothiazide could preserve bone mineral density in people with a high risk of osteoporosis [68], and a systematic review and meta-analysis of RCTs showed that thiazide diuretics could reduce fracture risk [79]
Zhang Y, Wang Y, 2023 (PMID: 37603265)	PSDs	Osteoarthritis	PSDs and aldosterone antagonists were associated with a lower risk of osteoarthritis	
Zhuang Z, 2024 (PMID: 38725159)	BBs; CCBs	Frailty	BBs and CCBs were potentially associated with reduced frailty risk	RCTs are not available
Longevity				
Fan B, 2024 (PMID: 38769606)	BBs; CCBs; vasodilators	Lifespan	BBs, CCBs, and vasodilators were related to longevity	RCTs are not available
Diseases of pregnancy				
Ardissino M, 2022 (PMID: 36064525)	BBs; CCBs	Pre-eclampsia or eclampsia; gestational diabetes; birthweight of the first child	CCB showed a protective effect against the risk of pre-eclampsia and eclampsia with no effect on gestational diabetes, or changes in birthweight of first child. However, BB may be associated with a reduction in birthweight	A systematic review and meta-analysis of RCTs reported that there is no reliable estimate of the effects of BBs and CCBs on pregnancy adverse outcomes, including pre-eclampsia [80]
Genitourinary diseases				
Zhao C, Feng JL, Deng S, 2023 (PMID: 37363097)	ACE inhibitors; BBs; CCBs; thiazide diuretics	Erectile dysfunction	No significant links were identified between the use of ACE inhibitors, BBs, CCBs, and thiazide diuretics and the risk of erectile dysfunction	A systematic review and meta-analysis of RCTs indicated that ACE inhibitors, BBs, CCBs, and thiazide diuretics have no impact on erectile dysfunction [67]

*ACE* Angiotensin-converting enzyme, *AD* Alzheimer’s disease, *ARBs* Angiotensin receptor blockers, *BBs* Beta-blockers, *CCBs* Calcium channel blockers, *CHD* Coronary heart disease, *eBMD* estimated heel bone mineral density, *eGFR* estimated glomerular filtration rate, *HDL* High-density lipoprotein, *IA* Intracranial aneurysms, *LDL* low-density lipoprotein, *PSDs* Potassium-sparing diuretics, *RCTs* Randomized controlled trials, *SAH* Subarachnoid hemorrhage, *TB-BMD* Total body bone mineral density, *TNF-alpha* Tumor necrosis factor alpha

## Discussion

This is the first systematic review on MR evaluating the effects of antihypertensive drug classes on various health outcomes. Our review found that antihypertensive drugs are beneficial for cardiovascular diseases. ACE inhibitors are protective for diabetes whereas BBs showed adverse effects. ACE inhibitors may increase the risk of schizophrenia, Alzheimer’s disease, and psoriasis but do not affect COVID-19 risk. ACE inhibitors and CCBs are beneficial for kidney function and immune function. CCBs showed a safe profile for disorders of pregnancy whereas BBs did not.

Our review highlights strong evidence of a protective role of antihypertensive drugs, specifically ACE inhibitors and CCBs, in CHD, stroke, post-stroke functional outcomes, diabetes, and kidney function, which is supported by evidence from RCTs [55–62], as well as national and international guidelines [81–84]. Although RCT evidence is lacking, strong MR evidence suggests an association between ACE inhibitors and increased schizophrenia risk in both Europeans and East Asians [32], highlighting the need for greater pharmacovigilance. Possible biological mechanisms can involve the central renin-angiotensin system, which affects inflammation and immunity that contribute to the development of schizophrenia [85]. MR also provides strong evidence that genetically predicted ARBs and thiazide diuretics have a protective effect on bone mineral density and fracture risk, supported by RCTs [68]. This could be explained by the role of thiazide diuretics in modulating calcium homeostasis by inhibiting thiazide-sensitive sodium chloride cotransporter in osteoblasts [86]. Hence, thiazide diuretics may be repurposed to improve bone health and lower the risk of fracture. Although there is MR evidence showing the potentially harmful effects of ACE inhibitors on colorectal cancer, of BBs on hepatocellular carcinoma, and of CCBs on prostate cancer, meta-analysis of RCTs consistently reported null [55, 64].

In the risk of bias assessment, we found most studies used different analytic methods, such as weighted median, weighted mode, MR-Egger, and MR-PRESSO. Alternative methods, such as MR Robust Adjusted Profile Score and contamination-mixture methods, were less frequently used. To strengthen the robustness of MR estimates, some studies used colocalization [32, 39, 42, 48, 53, 54], replication using different traits such as diastolic blood pressure and pulse pressure [17, 32, 44, 53, 54], and applied control outcomes [16, 28, 29, 31, 38–44, 48, 50]. Most studies were conducted in the European population while five studies considered the potential differences by ethnicity, which addressed research gaps in the safety and effectiveness of antihypertensive drugs in less frequently studied populations such as East Asians [31, 32, 39, 42, 48]. In addition, three studies used sex-specific genetic instruments to evaluate sex-specific outcomes [40, 41, 53].

### Strengths and limitations

In situations where there is a lack of RCTs for certain outcomes, this systematic review of MR findings summarized evidence regarding the effects of antihypertensive drugs with a formal evaluation of the strength of evidence and quality assessment. However, there are several limitations. First, in the absence of a standard MR protocol to assess quality, our evaluation was based on the STROBE-MR guideline [22] and previously proposed criteria [87]. Among the significant associations, the associations with hepatocellular carcinoma, peripheral artery disease, disorders during pregnancy, erectile dysfunction, kidney stones, renal and immune biomarkers, longevity, mental disorders, Parkinson’s disease, Huntington’s disease, osteoarthritis, and risk of fracture are based on only one MR study at the time of conducting this review. Although we assessed the quality of these studies, given the varying quality from “low-to-moderate” to “high,” we are conservative in drawing conclusions. Second, we did not meta-analyze MR results for the same exposure-outcome pairs because they used the same outcome data sources or outcome data with large overlapping samples, or used different exposure traits, or did not clarify exposure units (Additional file 2: Table S6). Third, we did not compare MR with studies from real-world settings because it is difficult to determine whether differences in results are due to study design differences or differences between target-mediated and non-target-mediated effects. Additionally, MR studies that use instrumental variables are mainly based on well-defined, known drug targets. However, drugs typically exhibit multi-target effects, including off-target effects deviating from known primary targets. Therefore, MR studies cannot detect off-target effects mediated by unknown targets that may exist in the real world. Future real-world studies are necessary to triangulate evidence on possible off-target effects of drugs mediated by unknown targets. Since MR and RCTs are both study designs that can provide causal estimates, we focused on the comparison between evidence from MR and RCTs. Fourth, the review mainly included ACE inhibitors, CCBs, BBs, and thiazide diuretics, which were the most often studied antihypertensive drug classes. Other classes were not often studied due to lack of genetic instruments. Fifth, given the limited availability of sex-specific genome-wide association studies, sex-specific genetic instruments for health outcomes are rare. Conducting sex-specific analyses in future MR research would be beneficial in closing the research gap and addressing the sex disparities. Sixth, evidence from RCTs is not available for some outcomes, so comparisons with MR evidence are not possible. Although MR and RCTs both provide causal estimates, the findings from the two study designs are sometimes inconsistent and their estimates may not be directly comparable. Particularly, drug target MR uses genetic instruments based on known targets to assess drug effects [88], while RCTs assess the effects of drugs via all targets. MR studies require a large sample size as the genetic instruments can only predict a small proportion of variation in the exposure [89], so MR studies may not have sufficient power. Lastly, the conclusions of this systematic review are generally applicable to Europeans, although five MR studies investigated the effects in East Asians [31, 32, 39, 42, 48], we did not find any available ancestry-specific RCTs on these outcomes, so we did not compare whether associations remain consistent across ethnicities. Evidence for other populations is still lacking.

## Conclusions

In summary, this systematic review on antihypertensive drugs summarizes evidence based on MR and offers valuable insights into the long-term effects of antihypertensive drugs with respect to various outcomes, including cardiovascular diseases, kidney health, diabetes and metabolic disorders, psychiatric and neurodegenerative diseases, cancer, infectious diseases, and immune function, and others to inform clinical decision-making.

## Supplementary Information

Additional file 1. Methods.

Additional file 2. Tables S1-S7. Table. S1– Criteria for evaluating the strength of evidence. Table. S2– Criteria for assessing risk of bias. Table. S3– Data extraction results. Table. S4– Strength of evidence evaluation results. Table. S5– Bias assessment results. Table. S6– Outcomes with more than two studies. Table S7– Literature research and screening results.

Additional file 3. Figures S1-S2. Fig. S1– Results of major antihypertensive drugs and other health conditions (continuous outcomes). Fig. S2– Results of other antihypertensive drug classes and diseases.

## Data Availability

No datasets were generated or analysed during the current study.

## Abbreviations

ACE: Angiotensin-converting enzyme
AD: Alzheimer’s disease
ARBs: Angiotensin receptor blockers
BBs: Beta-blockers
CCBs: Calcium channel blockers
CI: Confidence interval
CHD: Coronary heart disease
MR: Mendelian randomization
MR-PRESSO: Mendelian randomization pleiotropy residual sum and outlier
RCTs: Randomized controlled trials
