# Supplementary material for: Systematic review of Mendelian randomization studies on antihypertensive drugs
Source: BMC Med. 2024 Nov 20;22:547. doi: 10.1186/s12916-024-03760-x (PMC11580643; doi:10.1186/s12916-024-03760-x)
Supplement: Supplementary file 1 — Additional file 1. Methods. [file 12916_2024_3760_MOESM1_ESM.docx]

**Title: Systematic review of Mendelian randomization studies on antihypertensive drugs**

**Additional file 1: Methods**

Search terms for MR studies:

(Mendelian randomization OR Mendelian randomisation OR genetic instrumental variable OR genetic instrument) AND (blood pressure OR antihypertensives OR antihypertensive drug OR ACE inhibitors OR angiotensin II receptor blockers OR calcium channel blockers OR alpha-adrenoceptor blockers OR adrenergic neuron blocking drugs OR beta-adrenoceptor blockers OR centrally acting antihypertensive drugs OR loop diuretics OR potassium-sparing diuretics and aldosterone antagonists OR renin inhibitors OR thiazides OR diuretics OR vasodilator antihypertensives)
