# Supplementary material for: Systematic review of Mendelian randomization studies on antihypertensive drugs
Source: BMC Med. 2024 Nov 20;22:547. doi: 10.1186/s12916-024-03760-x (PMC11580643; doi:10.1186/s12916-024-03760-x)

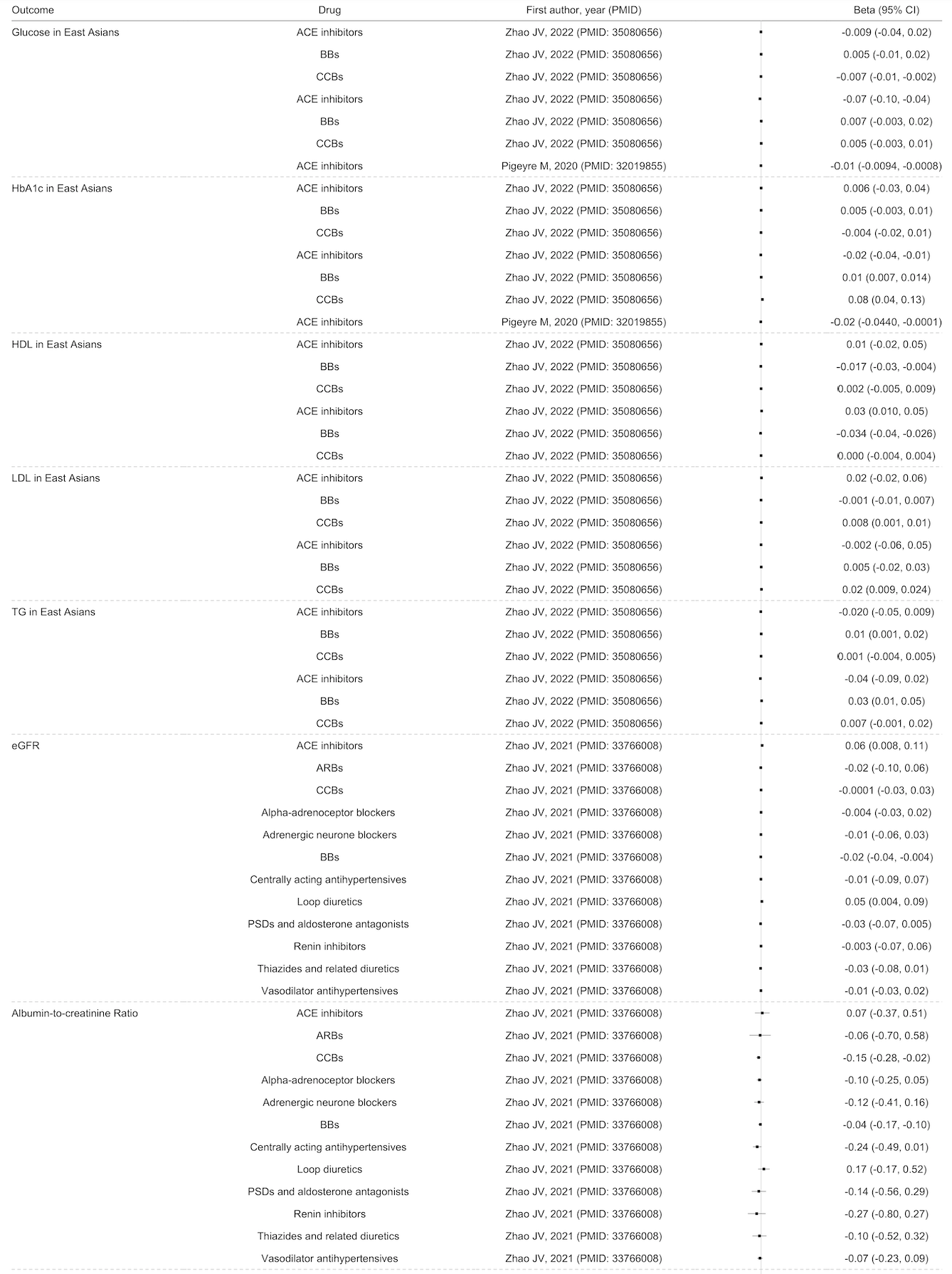
Figure S1. Results of major antihypertensive drugs and other health conditions (continuous outcomes)


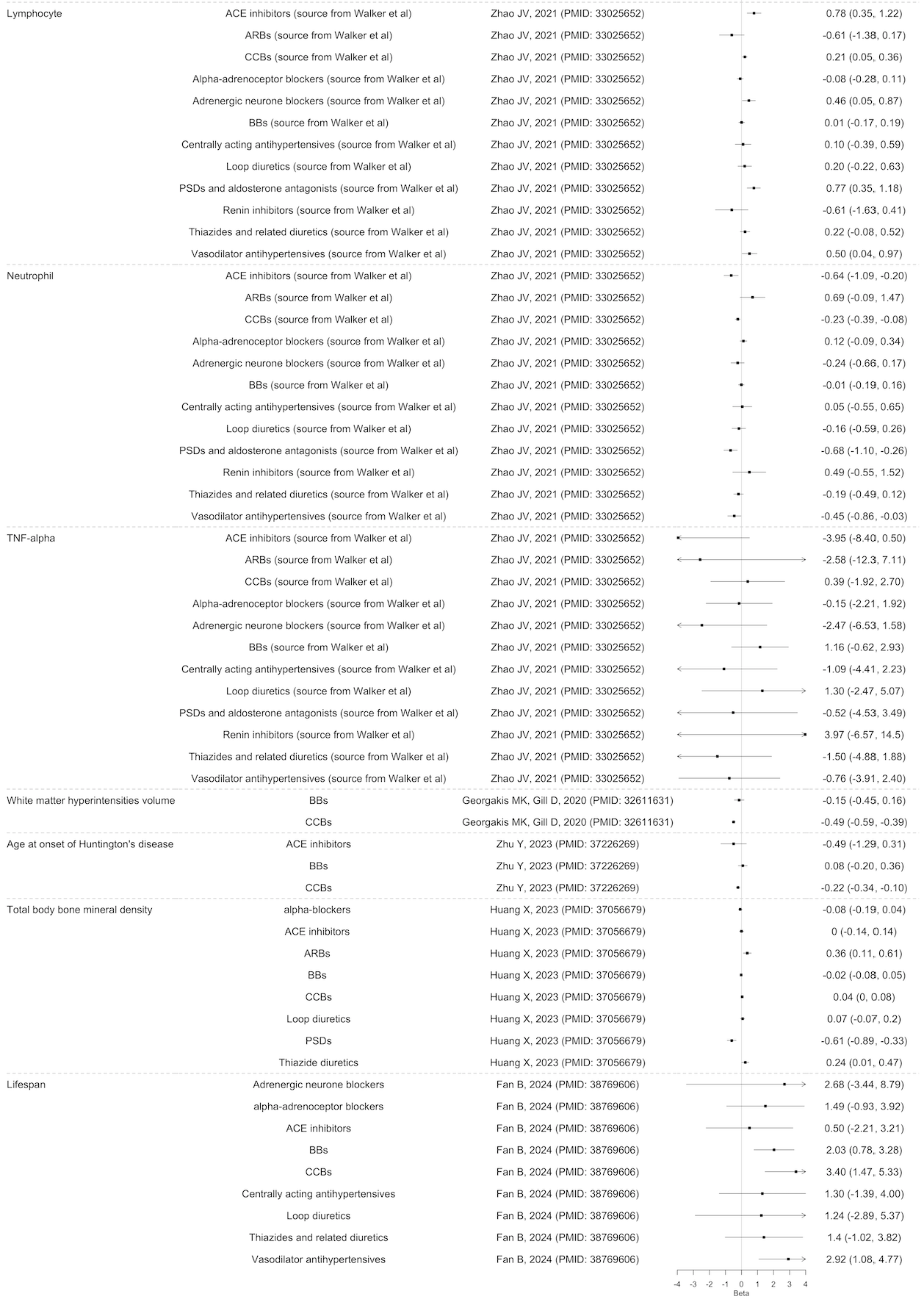


Figure S2. Results of other antihypertensive drug classes and diseases


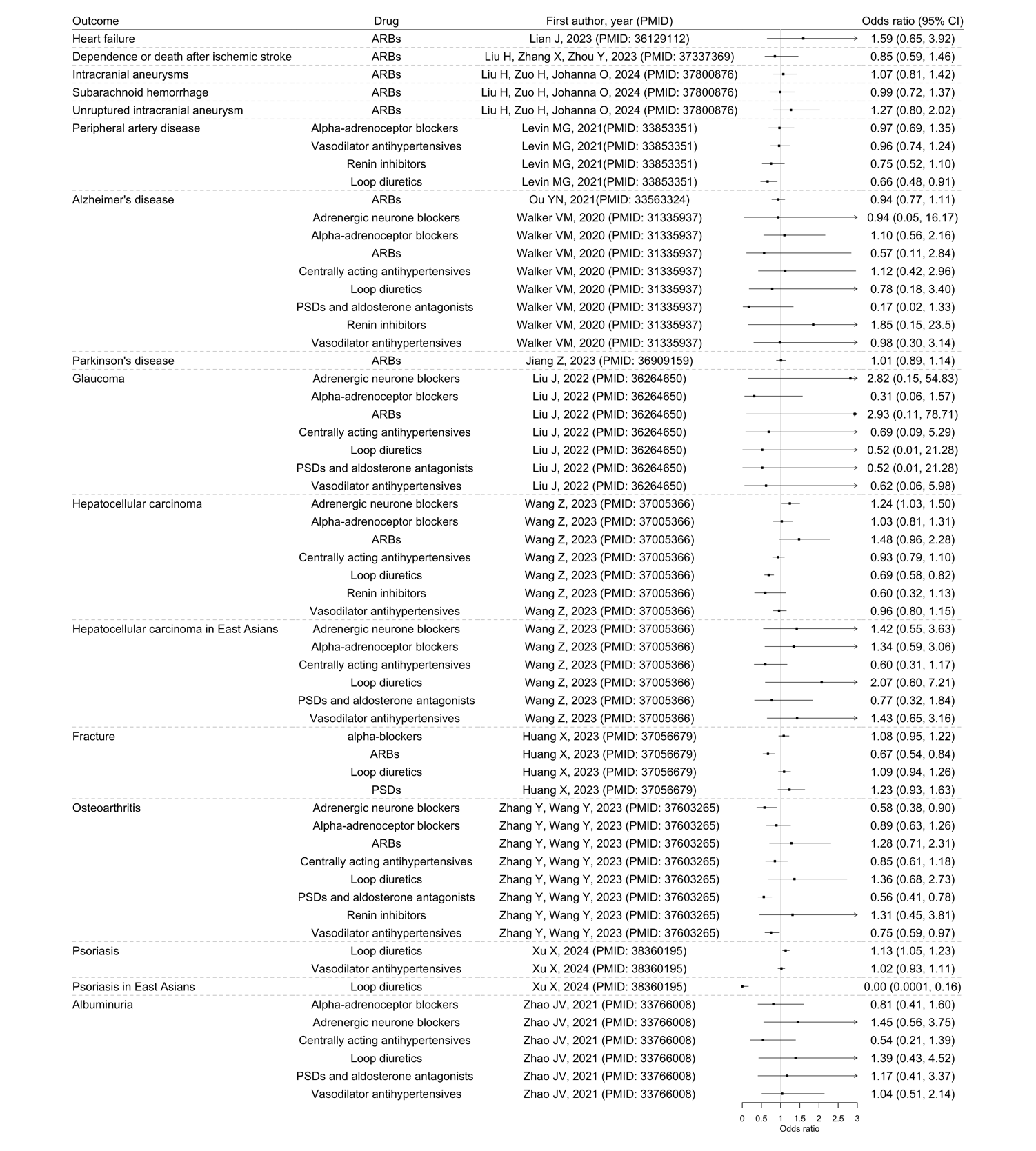

Supplement: Supplementary file 3 — Additional file 3. Figures S1-S2. Fig. S1– Results of major antihypertensive drugs and other health conditions (continuous outcomes). Fig. S2– Results of other antihypertensive drug classes and diseases. [file 12916_2024_3760_MOESM3_ESM.docx]
